# Erythropoietin produced by genetic-modified NIH/3T3 fibroblasts enhances the survival of degenerating neurons

**DOI:** 10.1002/brb3.356

**Published:** 2015-06-03

**Authors:** Yi-Chin Li, Shiu-Jau Chen, Chung-Liang Chien

**Affiliations:** 1 Department of Anatomy and Cell Biology, College of Medicine, National Taiwan UniversityTaipei, Taiwan; 2 Department of Medicine, Mackay Medical CollegeNew Taipei, Taiwan

**Keywords:** EPO, neurodegeneration, neuronal survival, NIH/3T3 fibroblasts

## Abstract

**Background:**

Erythropoietin (EPO) has potent neuroprotective effects. The short-term delivery of high-dose EPO seemed to improve patients’ neuromuscular functions; however, excessive EPO resulted in systematically high hematocrit and thrombotic risk. In our study, we established a cellular material for future *in vivo* studies of neurodegenerative diseases based on EPO provided regionally at a nontoxic level.

**Methods:**

A mouse EPO cDNA was subcloned into the pCMS-EGFP vector and transfected into NIH/3T3 fibroblasts to design a biological provider that can regionally release EPO for the treatment of neurological diseases. After G418 selection, a stable EPO-overexpressing cell line, EPO-3T3-EGFP, was established. To further confirm the neuroprotective abilities of secreted EPO from EPO-3T3-EGFP cells, a cell model of neurodegeneration, PC12-INT-EGFP, was applied.

**Results:**

The expression level of *EPO* was highly elevated in EPO-3T3-EGFP cells, and an abundant amount of EPO secreted from EPO-3T3-EGFP cells was detected in the extracellular milieu. After supplementation with conditioned medium prepared from EPO-3T3-EGFP cells, the survival rate of PC12-INT-EGFP cells was significantly enhanced. Surprisingly, a fraction of aggregated cytoskeletal EGFP-tagged *α*-internexin in PC12-INT-EGFP cells was disaggregated and transported into neurites dynamically. The immunocytochemical distribution of IF proteins, including NF-M, phosphorylated-NF-M, and the *α*-INT-EGFP fusion protein, were less aggregated in the perikaryal region and transported into neurites after the EPO treatment.

**Conclusion:**

The established EPO-overexpressing NIH/3T3 cell line, EPO-3T3-EGFP, may provide a material for future studies of cell-based therapies for neurodegenerative diseases via the secretion of EPO on a short-term, high-dose, regional basis.

## Introduction

Erythropoietin (EPO), a well-known hematopoietic cytokine, is pleiotropic which exerts a distinctive neuroprotection effect, and plays a strong role in neuronal survival (Sirén et al. 2001; Ghezzi and Brines 2004; Byts and Sirén 2009; Arcasoy 2010; Subiros et al. 2012). In recent decades, there has been a growing interest among researchers worldwide to develop potential therapeutic strategies via the application of EPO in patients with neurological diseases, such as cerebral ischemia, traumatic brain injury, and neurodegenerative and neuroinflammatory diseases (Lewczuk et al. 2000; Sirén et al. 2001; Ghezzi and Brines 2004; Byts and Sirén 2009; Subiros et al. 2012; Broxmeyer 2013). Although the short-term application of high-dose EPO seems to improve neuromuscular functions in patients with neurological diseases, excessive EPO contributes to high hematocrit systematically and the elevation of thrombotic risk in these patients (Sirén et al. 2001; Vesey et al. 2004; Kirkeby et al. 2008; McPherson and Juul 2010; Meng et al. 2011). In our study, we addressed a new biological material for neurological diseases based on EPO provided regionally at a nontoxic level; for instance, cell-based therapy.

Stem cell therapy has been proposed as a potential candidate for treating neurological diseases by inducing pluripotent stem cells into differentiated neurons (Alvarez et al. 2010; Yoo et al. 2013); however, many unaddressed risks remain in this context. A high risk of tumorigenesis transformed from the transplantation of differentiated neurons remains an obstacle to the clinical usage of stem cell therapies (Shih et al. 2007; Knoepfler 2009). To seek another approach for cell-based therapies, we suggested the differentiated fibroblasts as an alternative choice (Chen et al. 2012).

Human primary kidney cells can be enriched for EPO-producing cells by magnetic-bead sorting system. After administration of this subpopulation into a chronic renal injury rat model, renal function and renoprotective effects were markedly improved (Yamaleyeva et al. 2012). One of our previous studies demonstrated that the implantation of 3T3 fibroblasts into the hippocampal striatum area of mice with intracerebral hemorrhage induces neurogenesis, reduces loss of brain tissue, and restores function (Chen et al. 2012). The transplanted 3T3 fibroblasts survived and functioned well within 2 weeks after the transplantation, and appeared less tumorigenic (Chen et al. 2012). Since the cell-based delivery of EPO to the damaged local brain area has not been demonstrated in the animal model of neurological disease, we considered to establish an EPO-overexpressing fibroblast cell line in our study as a material which might represent a short-term, high-dose, and regional therapeutic concept for future in vivo study on the treatment of neurological diseases.

To test the bioactivities of secreted EPO, a cell model of neurodegeneration, PC12-INT-EGFP cells, which was established in our laboratory, was used in this study (Chien et al. 2005). *α*-internexin is a member of the family of neuronal intermediate filament (IF) proteins and coexists with neurofilament triplet proteins that are designated as NF-L, NF-M, and NF-H for low- (L), medium- (M), and high- (H) molecular-weight subunits (Yuan et al. 2006). In the presence of overexpression of the *α*-internexin-enhanced green fluorescent protein fusion protein (*α*-INT-EGFP) in PC12 cells, PC12-INT-EGFP cells showed progressive neuronal death because of the abnormal accumulation of overexpressed *α*-internexin (Chien et al. 2005; Lee et al. 2012). Neuronal aggregation is a general characteristic found in many neurodegenerative diseases, such as neuronal intermediate filament inclusion disease (NIFID), amyotrophic lateral sclerosis (ALS), Alzheimer’s diseases, Parkinson’s disease, and Huntington’s disease (Ross and Poirier 2004; Ching and Liem 2006; Perrot and Eyer 2013). Hence, we intended to test whether EPO secreted by EPO-overexpressing NIH/3T3 fibroblasts enhances the survival of degenerating PC12-INT-EGFP cells.

The aims of our study were to establish an EPO-overexpressing NIH/3T3 fibroblast cell line and to identify possible neuroprotective effects of secreted EPO to provide a potential material for studies of cell-based therapy in animal models of neurological disorders.

## Materials and Methods

### EPO-pCMS-EGFP plasmid construction

The clone with a full-length 579 bp mouse EPO cDNA was purchased from Sino Biological Inc. (Beijing, China). The coding region of mouse EPO was amplified by PCR using designed restriction enzyme sites: *EcoRI* at the 5′ end and *SalI* at the 3′ end. The primers used to clamp the mouse EPO cDNA were: (forward primer) *5′–GAA TTC ATG GGG GTG CCC GAA–3′* and (Reverse primer) *5′–GTC GAC TC ACC TGT CCC CTC T–3′*. The PCR-modified mouse EPO cDNA was subcloned into pCMS-EGFP (Clontech, Palo Alto, CA) to obtain the nonfusion coding sequence of EGFP. After confirmation by DNA sequencing, plasmids carrying a 100% correct sequence of the mouse EPO cDNA (RefSeq: NM_007942.2; http://www.ncbi.nlm.nih.gov/nucleotide, RRID: nlx_84100) were constructed and named EPO-pCMS-EGFP.

### Cell culture and differentiation

The NIH/3T3 cell line (ATCC® CRL-1658™) was cultured in Dulbecco’s Modified Eagle’s Medium (DMEM) supplemented with 10% fetal bovine serum (FBS), 1% nonessential amino acids (NEAAs), and 1× antibiotic/antimycotic solution (Invitrogen, Carlsbad, CA).

The PC12-INT-EGFP cell line was cultured in DMEM supplemented with 7.5% heat-inactivated FBS, 7.5% heat-inactivated horse serum (Invitrogen), and 1× antibiotic/antimycotic solution (Invitrogen), and was maintained by 300 *μ*g/mL G418 sulfate (Millipore, Darmstadt, Germany). The PC12-INT-EGFP cell line was established and characterized well by Chien et al. (2005). Neuronal differentiation was induced using 100 ng/mL of nerve growth factor (NGF; R&D Systems, Minneapolis, MN) for 6 days before treatments with conditioned media.

Cells were seeded on culture dishes (Corning, NY) and maintained in a 37°C humidified incubator with 5% CO_2_. The medium was replaced with fresh medium every 2 days.

### Plasmid transfection and stable cell clone selection

NIH/3T3 cells (1 × 10^5^) were seeded on 35 mm culture dishes 1 day prior to plasmid transfection. Lipofectamine® LTX & PLUS™ reagents (Invitrogen) were used. NIH/3T3 cells were transfected with a plasmid mixture of 3 *μ*g of EPO-pCMS-EGFP and pDsRed-monomer-N1 or pCMS-EGFP and pDsRed-monomer-N1 (Clontech) separately at a ratio of 5:2. pDsRed-monomer-N1 with a kanamycin/neomycin resistance gene was cotransfected for G418 stable clone selection. Transfection procedure was in accordance with the instruction manual. Subsequently, cells were incubated in a humidified incubator with 5% CO_2_ at 37°C overnight, and changed to respective 3T3 growth medium. G418 sulfate (800 *μ*g/mL) was applied to the transfected cells for stable clone selection. After 14 days of G418 selection, surviving cell colonies with strong green fluorescence were picked under an inverted fluorescence microscope (DM IRME HC, Leica, Wetzlar, Germany). An EPO-3T3-EGFP stable cell clone and our experiment control, the 3T3-EGFP stable cell clone, were isolated for use in subsequent studies.

### Reverse transcription PCR (RT–PCR)

Cells were harvested and rinsed twice with cold Dulbecco’s Phosphate-Buffered Saline (DPBS). Total cellular RNA was extracted using the MaestroZol™ RNA PLUS Extraction Reagent (OmicsBio, Taipei City, Taiwan), according to the manufacturer’s instructions. The ThermoScript™ RT–PCR System (Invitrogen) was used for cDNA synthesis, according to the manual provided by the manufacturer.

Semiquantitative PCR was performed using the SapphireAmp® Fast PCR Master Mix (TAKARA, Shiga, Japan). The forward primer used for detecting the expression of *EPO* was *5′–TTG CTA CTG ATT CCT CTG GGC–3′*, and the reverse primer was *5′–GTG AGT GTT CGG AGT GGA GC–3′*. The expression level of the glyceraldehyde-3-phosphaate dehydrogenase (*GAPDH*) gene was used as the internal control. The forward primer used for the detection of *GAPDH* was *5′–AAC TTT GGC ATT GTG GAA GG–3′*, and the reverse primer was *5′–ACA CAT TGG GGG TAG GAA CA–3′*. The PCR reaction was processed at 94°C for 1 min to denature the cDNA templates, followed by 45 amplification cycles (denaturation at 98°C for 5 sec, annealing at 57°C for EPO primers and 55°C for GAPDH primers for 5 sec, and extension at 72°C for 10 sec), and ended by a terminal extension at 72°C for 2 min. Subsequently, PCR products were analyzed by 1% agarose gel electrophoresis.

### Quantitative real-time PCR (Q-PCR)

Q-PCR was performed by using KAPA SYBR® FAST qPCR kit (KAPABiosystems, Wilmington, MA). Samples were run and detected on an Mx300P Real-time PCR system (Stratagene, La Jolla, CA) in a final reaction volume of 10 *μ*L. The thermocycling procedure was as follows: enzyme activation at 95°C for 3 min, 45 amplification cycles (denaturation at 95°C for 3 sec, annealing at 55°C for 20 sec, and extension at 72°C for 1 sec), and a final three-step dissociation (95°C for 1 min, 55°C for 30 sec, and 95°C for 30 sec). The expression level of *EPO* in each group was normalized to that of *GAPDH*. The following primers were used for Q-PCR: EPO, *5′–TTG CTA CTG ATT CCT CTG GGC–3′* and *5′–GTG AGT GTT CGG AGT GGA GC–3′*; and GAPDH, *5′–AAC TTT GGC ATT GTG GAA GG–3′*, and *5′–ACA CAT TGG GGG TAG GAA CA–3′*. The resulting data were further analyzed using a two-tailed *t*-test (paired) and were plotted using GraphPad Prism® 4.0 (GraphPad Software Inc., La Jolla, CA, http://www.graphpad.com/, RRID: rid_000081).

### Western blot analysis

For protein extraction, cells were harvested and rinsed twice with cold DPBS (Invitrogen). Cell pellets were homogenized in protein lysis buffer (Cell Signaling, Danvers, MA). Cell lysates were denatured at 97°C for 10 min in 1× sample buffer. For culture supernatants, 10 *μ*L out of a total of 10 mL of cultured medium was boiled with 1× sample buffer. All samples were stored at −20°C before analysis. Proteins (40 *μ*g/per lane) were loaded onto 12% SDS–PAGE. Proteins were transferred to a PVDF membrane. After blocking with 5% nonfat dry milk dissolved in TBS-T (0.1% Tween-20 in 1× TBS) for 40 min at room temperature, the membrane was hybridized with primary antibodies against EPO (1:200; Santa Cruz, Dallas, TX; Cat# sc-7956, RRID: AB_2098408), EGFP (1:2000; Millipore, Cat# MAB3580, RRID: AB_94936), and GAPDH (1:5000; Millipore, Cat# MAB374, RRID: AB_2107445) in the blocking buffer and incubated at 4°C overnight. After washing in TBS-T, the membrane was incubated with horseradish peroxidase-conjugated secondary antibodies diluted in TBS-T at room temperature for 1 h. The membrane was washed again and protein signals were developed using the SuperSignal West Femto Chemiluminescent Substrate (Thermo, Rockford, IL) and detected using a G:BOX/iChemi XL imager (Syngene, Cambridge, UK).

### Enzyme-linked Immunosorbent Assay (ELISA)

The concentration of EPO in cell lysates (40 *μ*g, nondenatured) was also quantitated by ELISA. In addition, for the measurement of culture supernatants, 3 × 10^5^ 3T3, 3T3-EGFP, and EPO-3T3-EGFP cells were seeded on 60 mm dishes on Day 0. Culture supernatants were collected after 24, 48, and 72 h. Cell number was also counted. The culture supernatants collected were centrifuged at 1500 rpm, 4°C for 10 min to discard the cell debris and were stored at −80°C. The Mouse Erythropoietin Quantikine ELISA Kit (R&D Systems) was used to obtain quantitative data regarding the amount of both cell lysates and secreted EPO in culture supernatants by following the manual supplied by the manufacturer. After the reaction was terminated using Stop Solution, optic density was read at 450 nm on a microplate reader (Ultrospec ® 3100 pro; Amersham Bioscience Corp., Piscataway, NJ).

### Functional assays of EPO in conditioned media

The bioactivity of EPO secreted from the EPO-overexpressing NIH/3T3 cell line was examined in a cell model of neurodegeneration, PC12-INT-EGFP cells. 3T3, 3T3-EGFP, and EPO-3T3-EGFP cells (1.6 × 10^6^ cells) were seeded in 10 cm dishes and cultured for 24 h. Culture supernatants were collected by sterile syringes and filtered through hydrophilic polyethersulfone membranes with a pore size of 0.22 *μ*m (Millipore) for subsequent functional assays. The concentration of secreted EPO in collected culture supernatants was also measured using the Mouse Erythropoietin Quantikine ELISA Kit (R&D Systems). After 6 days of NGF induction, PC12-INT-EGFP cells were supplemented with conditioned media (50% v/v), which combined half of the fresh medium for PC12 cells and half of the collected culture supernatants, as mentioned previously. Moreover, 10 IU/mL of recombinant human EPO (hrEPO; specific activity, 115,336 IU/mg P; Millipore) was applied as the positive control. PC12 cells treated with fresh medium alone were used as the vehicle group. A cell viability assay, 48 h live-cell imaging, and immunocytochemistry were performed on PC12-INT-EGFP cells for testing the bioactivity of the secreted EPO.

### Cell-viability assay: propidium iodide (PI)/Hoechst 33342 costaining

PC12-INT-EGFP cells (5 × 10^3^ cells) were seeded in 4-well chamber slides (Millipore) coated with 50 *μ*g/mL poly-d-lysine. After 6 days of NGF induction, PC12-INT-EGFP cells were supplemented with conditioned media (50% v/v). Conditioned media were removed after 48 h of treatment. PC12-INT-EGFP cells were incubated in a mixture of the nuclear dyes Hoechst 33342 (1:3500; stock concentration, 10 *μ*g/mL; Invitrogen) and PI (1:200; stock concentration, 1 *μ*g/mL; Sigma-Aldrich, St. Louis, MO) diluted in fresh medium for PC12 cells for 40 min, followed by aspiration of the medium. Cells were washed in DPBS and fixed with 4% paraformaldehyde (PFA) for 15 min at room temperature. After washing with DPBS, specimens were mounted with Fluoro-Gel (Electron Microscopy Sciences, Hatfield, PA). Fluorescent images were acquired using a Leica DMR fluorescence microscope and a TCS SP5 confocal microscope (Leica).

#### The cell-survival rate was calculated as follows



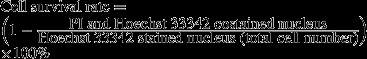


Data were analyzed statistically using one-way ANOVA and plotted using GraphPad Prism® 4.0 (GraphPad Software Corp.).

#### Live-cell imaging via time-lapse microscopy

PC12-INT-EGFP cells (5 × 10^4^ cells) were seeded in a 6-well plate. After 6 days of NGF induction, conditioned media (50% v/v) were used to supplement the cultures. Live-cell images of PC12-INT-EGFP cells with different treatments were recorded every 20 min for 48 h using the Real-Time Cultured Cell Monitoring system CCM-1.4Z (ASTEC Co. Ltd, Fukuoka, Japan).

#### Immunocytochemistry

PC12-INT-EGFP cells (2 × 10^3^ cells) were seeded on cover slips coated with 50 *μ*g/mL of poly-d-lysine in 24-well culture plates. After 6 days of NGF induction, conditioned media (50% v/v) were used to supplement cultures for 24 h. Cells were then fixed with 4% PFA for 15 min at room temperature and washed with PBS. After blocking in blocking buffer (3% FBS in PBS-T) for 30 min, cells were hybridized with primary antibodies against NF-M (1:100; Sigma-Aldrich, Cat# N5264, RRID: AB_477278), p-NF-M (1:100; Millipore, Cat# 05-774, RRID: AB_309985), and EGFP (1:200; Millipore, Cat# AB3080, RRID: AB_91337) in blocking buffer at 4°C overnight. After the samples were washed with PBS-T (0.1% Triton X-100 in PBS), cells were incubated with fluorescent-labeled secondary antibodies (1:200; anti-mouse IgG-rhodamine, Sigma-Aldrich, Cat# T5268, RRID: AB_261693 or anti-rabbit IgG-FITC, Sigma-Aldrich, Cat# F9006, RRID: AB_259787) and Hoechst 33342 (1:1000; stock concentration, 10 *μ*g/mL; Invitrogen) for 1 h, followed by washing with PBS-T. Finally, specimens were mounted on microscope slides with Fluoro-Gel (Electron Microscopy Sciences). Fluorescent images were acquired using a TCS SP5 confocal microscope (Leica).

## Results

### Characterization of EPO in the 3T3, 3T3-EGFP, and EPO-3T3-EGFP stable cell lines

To obtain a constitutively EPO-overexpressing cell clone, 800 *μ*g/mL of G418 was applied for 14 consecutive days for stable clone selection after the cotransfection of EPO-pCMS-EGFP and pDsRed-monomer-N1 into NIH/3T3 cells. The established EPO-3T3-EGFP stable clone, with strong green fluorescence, was observed under an inverted fluorescence microscope, and the experimental control, 3T3-EGFP stable cell clone, was also isolated for subsequent studies (Fig. S1A). There was no significant difference in morphology and cell doubling time among the 3T3, 3T3-EGFP, and EPO-3T3-EGFP cell lines.

**Figure 1:**
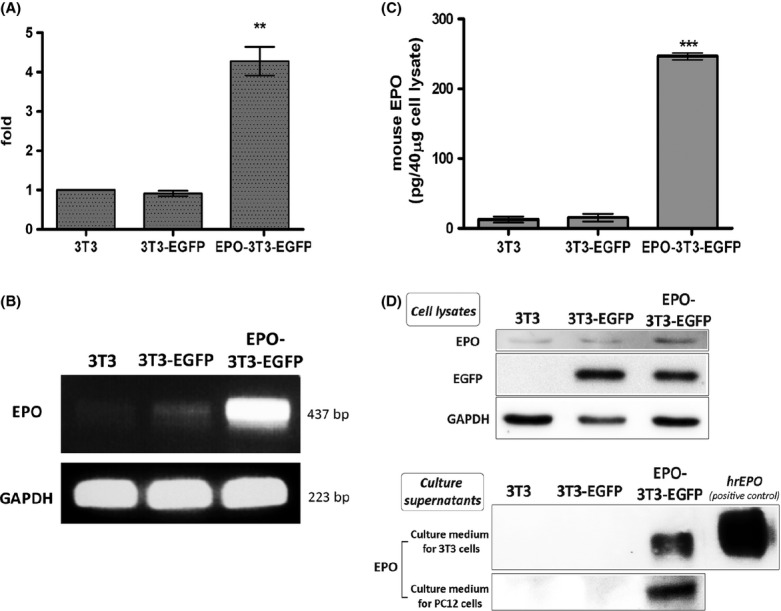
Characterization of *EPO* expression in the 3T3, 3T3-EGFP, and EPO-3T3-EGFP stable cell lines. Q-PCR (A) and RT–PCR (B) analyses of EPO RNA expression in the 3T3, 3T3-EGFP, and EPO-3T3-EGFP stable cell lines demonstrate that the EPO expression levels were highly elevated in EPO-3T3-EGFP cells. The expression level of EPO in the EPO-3T3-EGFP cell line is 4.27-fold higher than that observed in the 3T3 and 3T3-EGFP cell lines (n = 3, **P <* *0.05, ***P <* *0.001, two-tailed *t*-test). GAPDH was used as the internal control. Increase in cytosolic EPO in the lysates of EPO-3T3-EGFP cells, as determined by ELISA (C) and western blot analysis (D). Abundant secretion of overexpressed EPO in the culture supernatants of EPO-3T3-EGFP cells was detected, not only in the medium for 3T3 cells but also in the medium for PC12 cells (D). GAPDH was applied as the loading control of samples.

To verify whether the transfected *EPO* gene was correctly overexpressed in EPO-3T3-EGFP cells, we examined the RNA level of EPO using both Q-PCR and RT–PCR analyses. The Q-PCR results revealed the relative levels of the EPO mRNA in each cell line (Fig.1A). The expression level of *EPO* in the EPO-3T3-EGFP cell line was 4.27-fold higher than that observed in the 3T3 and 3T3-EGFP cell lines (*n* = 3, **P <* *0.05). RT–PCR also confirmed that the EPO-3T3-EGFP cell group expressed the transfected EPO cDNA (a 437 bp PCR product) at high levels (Fig.1B).

Next, we determined the protein levels of overexpressed EPO by ELISA and western blotting, to verify the presence of EPO in nontransfected and transfected NIH/3T3 cells. An increase of cytosolic EPO was observed in the EPO-3T3-EGFP cell group, whereas endogenous EPO was scarcely detected in the 3T3 and 3T3-EGFP cell groups (Fig.1C and D). Our ELISA data showed a significant increase in cytosolic EPO (246 ± 11.07 pg/40 *μ*g of cell lysate, mean ± SD; paired *t*-test, ***P <* *0.001) compared with 3T3 cells (12.68 ± 10.06 pg/40 *μ*g of cell lysate) and 3T3-EGFP cells (15.31 ± 12.30 pg/40 *μ*g of cell lysate). Similar patterns were observed using immunocytochemistry staining (Fig. S1B). In addition, extracellular EPO secretion from 3T3, 3T3-EGFP, and EPO-3T3-EGFP cells was analyzed by western blotting (Fig.1D). Secreted EPO was detected only in the EPO-3T3-EGFP groups, whether the cells were cultured in the medium for NIH/3T3 cells or in the medium for PC12 cells.

Taken together, our results of the examination of *EPO* expression levels indicate that the RNA expression levels in the EPO-3T3-EGFP cell line were significantly higher than they were in the 3T3 and 3T3-EGFP cell lines. Increased cytosolic EPO and secreted EPO were observed in the EPO-overexpressing NIH/3T3 cell line, EPO-3T3-EGFP.

### Concentration of secreted EPO in the culture supernatants from 3T3, 3T3-EGFP, and EPO-3T3-EGFP cells

To quantify the amount of EPO secreted from 3T3, 3T3-EGFP, and EPO-3T3-EGFP cells, we collected their culture supernatants and performed ELISA. Cells (3 × 10^5^) were seeded on Day 0, and culture supernatants were collected for 3 consecutive days (24, 48, and 72 h). The statistical data presented in Table 2010 showed that the level of EPO secreted from EPO-3T3-EGFP cells (4428.6 ± 156.3 pg/mL (mean ± SD) at 24 h; 11874.6 ± 724.1 pg/mL at 48 h; and 23888.8 ± 737.8 pg/mL at 72 h) was significantly higher than that secreted from 3T3 cells (undetectable at 24 and 48 h; 18.2 ± 31.5 pg/mL at 72 h) and 3T3-EGFP cells (undetectable at 24 h; 18.2 ± 31.5 pg/mL at 48 h; 34.4 ± 29.9 pg/mL at 72 h). There was no significant difference in cell doubling time among the groups.

**Table 1:** Quantification of erythropoietin (EPO) secreted from the 3T3, 3T3-EGFP, and EPO-3T3-EGFP stable cell lines using an Enzyme-Linked Immunosorbent Assay (ELISA).

	Number of hour(s) of EPO secretion (mean ± SD pg/mL, *n* = 3)		
Cell lines	24 h	48 h	72 h
3T3	–1	–1	18.2 ± 31.5
3T3–EGFP	–1	18.2 ± 31.5	34.4 ± 29.9
EPO-3T3-EGFP	4428.6^2^ ± 156.3	11874.6^2^ ± 724.1	23888.8^2^ ± 737.8

1 EPO was undetectable in the culture supernatants.

2 Highly significant amount of secreted EPO was detected in the culture supernatants compared with the 3T3 and 3T3-EGFP cell groups (one-way ANOVA, *P *<* *0.001).

Our ELISA results indicated that EPO was secreted very rarely into the extracellular milieu from nontransfected NIH/3T3 cells and the experimental control group, 3T3-EGFP cells. However, in the case of the EPO-3T3-EGFP cell line, EPO was abundantly secreted into the extracellular milieu. This evidence suggests that the EPO overexpressed from EPO-3T3-EGFP cells may be functional extracellularly.

### Cell viability of PC12-INT-EGFP cells after conditioned media treatments for 48 h

To examine the bioactivity of the secreted EPO, we supplemented the *α*-internexin-overexpressing PC12 cell line, PC12-INT-EGFP cells, with conditioned media (50% v/v) on Day 6 after NGF induction. The level of secreted EPO was 5.40 ± 1.36 ng/mL (mean ± SD, *n* = 5) in the culture supernatants collected from EPO-3T3-EGFP cells and was undetectable in those collected from 3T3 and 3T3-EGFP cells, as assessed by ELISA. hrEPO (10 IU/mL) was also applied as the positive control. The functional bioactivity of secreted EPO was determined using a cell viability assay, 48 h live-cell imaging, and immunocytochemistry in PC12-INT-EGFP cells after conditioned media treatments.

**Figure 2:**
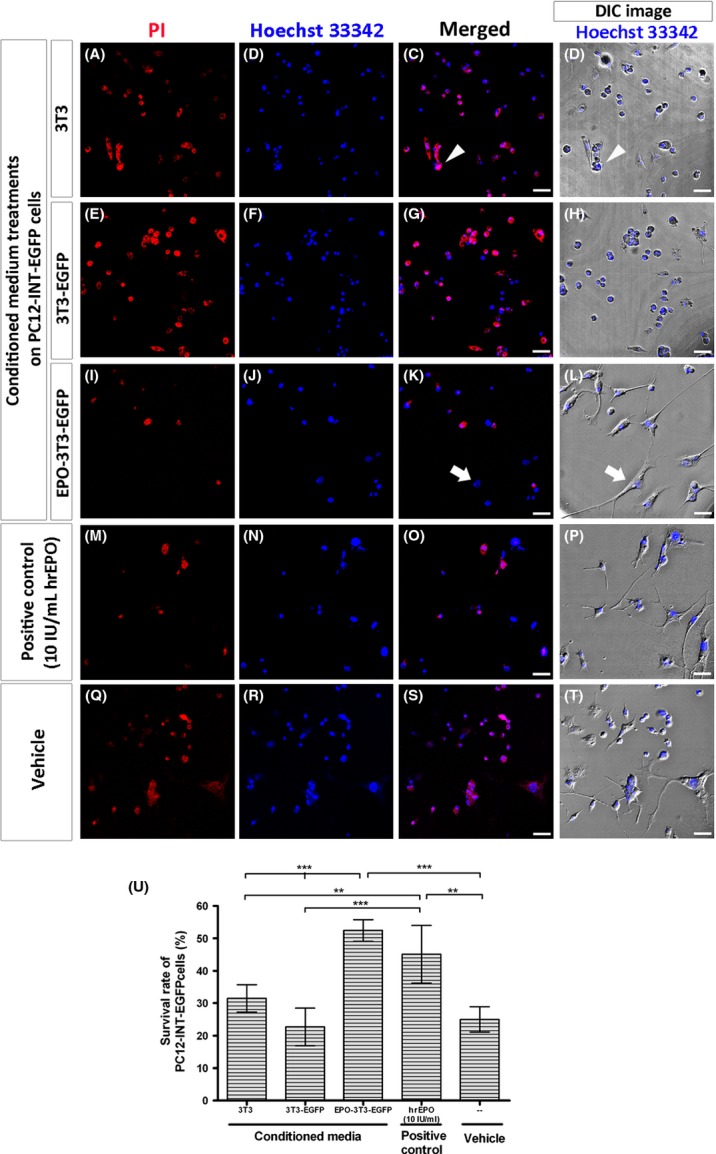
Cell-viability assay for cultured PC12-INT-EGFP cells after 3T3, 3T3-EGFP, and EPO-3T3-EGFP-conditioned media treatments for 48 h. The cell viability assay for the survival of PC12-INT-EGFP cells was assessed using PI/Hoechst 33342 costaining of cellular nuclei after supplementation with 3T3 (A–D), 3T3-EGFP (E–H), and EPO-3T3-EGFP (I–L)-conditioned media (50% v/v), or with 10 IU/mL of hrEPO (M–P) as well as the vehicle (Q–T) for 48 h after 6 days of NGF induction. The DIC images merged with Hoechst 33342 (D, H, L, P, and T) demonstrated that, morphologically, dying PC12-INT-EGFP cells appeared to be rather spherical (C and D, arrowhead) compared with the surviving cells, which exhibited a flattened neuronal pattern (K and L, arrow). Scale bars = 40 *μ*m. (U) The survival rate of PC12-INT-EGFP cells supplemented with EPO-3T3-EGFP-conditioned medium (52.46 ± 3.34%, mean ± SD, *n* = 4) was significantly enhanced compared with that observed in the groups supplemented with 3T3 (31.49 ± 4.22%) and 3T3-EGFP-(22.69 ± 5.79%)-conditioned media, or with the vehicle (25.03 ± 3.88%). hrEPO (10 IU/mL) was used as a positive control. Supplementation with 10 IU/mL of hrEPO led to a significant increase in the survival rate of PC12-INT-EGFP cells (45.12 ± 8.88%; one-way ANOVA, **P <* *0.01, ***P <* *0.001).

As PC12-INT-EGFP cells progressively underwent cell death after NGF induction (Chien et al. 2005), we wanted to assess whether the neuronal survival rate was increased after supplementation with EPO. The cell viability assay for the survival of PC12-INT-EGFP cells was assessed using PI/Hoechst 33342 nuclear costaining after supplementation with 3T3 (Fig.2A–D), 3T3-EGFP (Fig.2E–H), and EPO-3T3-EGFP (Fig.2I–L) conditioned media and 10 IU/mL of hrEPO (Fig.2M–P) for 48 h; we also analyzed the vehicle group (Fig.2Q–T). The DIC images showed that, morphologically, dying PC12-INT-EGFP cells, which were double-labeled with PI and Hoechst 33342 appeared to be rather spherical (Fig.2C and D, arrowhead) compared with the surviving cells, which exhibited a flattened neuronal pattern (Fig.2K and L, arrow).

The survival rate of PC12-INT-EGFP cells after different conditioned medium treatments was calculated for four repeated sets (*n* = 4), as shown in Fig.2U. The survival rate of PC12-INT-EGFP cells supplemented with EPO-3T3-EGFP-conditioned medium (52.46 ± 3.34%) for 48 h was significantly increased compared with that of groups supplemented with 3T3 (31.49 ± 4.22%) and 3T3-EGFP (22.69 ± 5.79%) conditioned media, or with the vehicle (25.03 ± 3.88%). Supplementation with 10 IU/mL of hrEPO led to a significant increase in the survival rate of PC12-INT-EGFP cells (45.12 ± 8.88%) compared with the groups supplemented with 3T3 and 3T3-EGFP-conditioned media, or with the vehicle (one-way ANOVA, **P <* *0.01, ***P <* *0.001).

Therefore, the functional significance of secreted EPO was suggested by the increase in the survival of the PC12-INT-EGFP neuronal cells after treatment with EPO-3T3-EGFP conditioned medium for 48 h.

### Live-cell imaging of PC12-INT-EGFP cells after conditioned media treatments

Time-lapse microscopy images were acquired every 20 min for 48 h to examine morphological changes and the dynamic distribution of the overexpressed green fluorescent *α*-internexin-EGFP fusion protein (*α*-INT-EGFP) in PC12-INT-EGFP cells after supplementation with 3T3, 3T3-EGFP, and EPO-3T3-EGFP conditioned media (50% v/v) or with 10 IU/mL of hrEPO on day 6 after NGF induction. Real-time images were transformed into video (10 images/sec), as shown in the Supplementary Videos. We found that a fraction of aggregated *α*-INT-EGFP proteins in PC12-INT-EGFP cells were redistributed and disaggregated in the groups supplemented with EPO-3T3-EGFP-conditioned medium or with 10 IU/mL of hrEPO.

Subsequently, we collected and compared green fluorescence images, as well as bright-field images merged with green fluorescence images at 0, 24, and 48 h of PC12-INT-EGFP cells after supplementation with 3T3 (Fig. S3A–C and S3a–c), 3T3-EGFP (Fig. S3D–F and S3d–f), and EPO-3T3-EGFP (Fig. S3G–I and S3g–i) conditioned media, or with 10 IU/mL of hrEPO (Fig. S3J–L and S3j–l) as well as the vehicle (Fig. S3M–O and S3m–o). We observed that PC12-INT-EGFP cells treated with conditioned medium from EPO-3T3-EGFP cells had a healthier appearance compared with the other groups. Interestingly, some aggregated green fluorescent *α*-INT-EGFP in PC12-INT-EGFP cells supplemented with conditioned medium from EPO-3T3-EGFP cells exhibited dynamic patterns of disaggregation, which were not observed in the groups supplemented with conditioned media from 3T3 and 3T3-EGFP cells or with the vehicle. Moreover, the disaggregating *α*-INT-EGFP exhibited a rearranged distribution and was transported into neurites (Fig. S3H, h, I, and i, arrows). A few PC12-INT-EGFP cells supplemented with 10 IU/mL of hrEPO showed similar patterns of disaggregation (Fig. S3L and l, arrows).

### Distribution patterns of NF-M, p-NF-M, and *α*-INT-EGFP in PC12-INT-EGFP cells at 24 h after conditioned media treatments

Our 48 h live-cell images might suggest that the EPO secreted from EPO-3T3-EGFP cells exhibited the bioactivity of rearranging and disaggregating the *α*-INT-EGFP overexpressed in PC12-INT-EGFP cells (Fig. S3G–I and S3g–i). Therefore, we wanted to investigate whether the secreted EPO also affected the distribution patterns of other IF proteins in PC12-INT-EGFP cells. Since *α*-internexin is highly related to NF-M axonal transportation and colocalizes with NF-M on a single neurofilament, as assessed by immunogold electron microscopy (Yuan et al. 2006), we focused mainly on the distribution of NF-M and its phosphorylated type, p-NF-M, together with *α*-INT-EGFP, respectively, by immunocytochemical analysis in this study.

**Figure 3:**
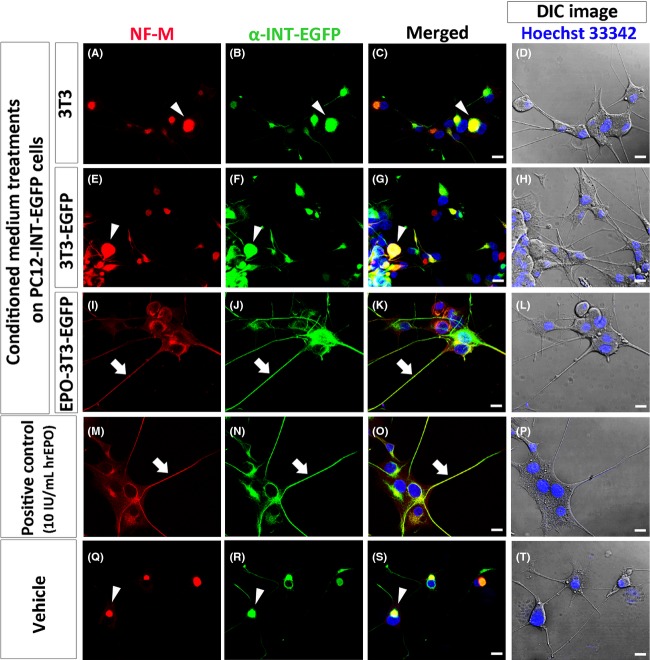
Distribution patterns of NF-M and *α*-INT-EGFP in PC12-INT-EGFP cells after 3T3, 3T3-EGFP, and EPO-3T3-EGFP conditioned media treatments. PC12-INT-EGFP cells were fixed 24 h after supplementation with 3T3 (A–D), 3T3-EGFP (E–H), and EPO-3T3-EGFP (I–L) conditioned media, or with 10 IU/mL of hrEPO (M–P) and the vehicle (Q–T). Highly aggregated NF-M and *α*-INT-EGFP was shown in the perikaryal region of the groups supplied with 3T3 (A–C), 3T3-EGFP (E–G) conditioned media and the vehicle group (Q–S), and the merged images indicate the colocalization of NF-M and *α*-INT-EGFP (C, G, S, arrowheads; yellow). In the group supplied with EPO-3T3-EGFP conditioned medium, NF-M (I, arrow) and *α*-INT-EGFP (J, arrow) in PC12-INT-EGFP cells were transported into neurites and appeared to be less aggregated in the perikaryal region. High colocalization between NF-M and *α*-INT-EGFP in neurites was indicated by merged images (K, arrow). Similar distribution patterns were also found in PC12-INT-EGFP cells supplemented with 10 IU/mL of hrEPO (M–O, arrow), which was used as the positive control. DIC images were taken in each group (D, H, L, P, T), to depict cellular morphology. Scale bars = 20 *μ*m.

**Figure 4:**
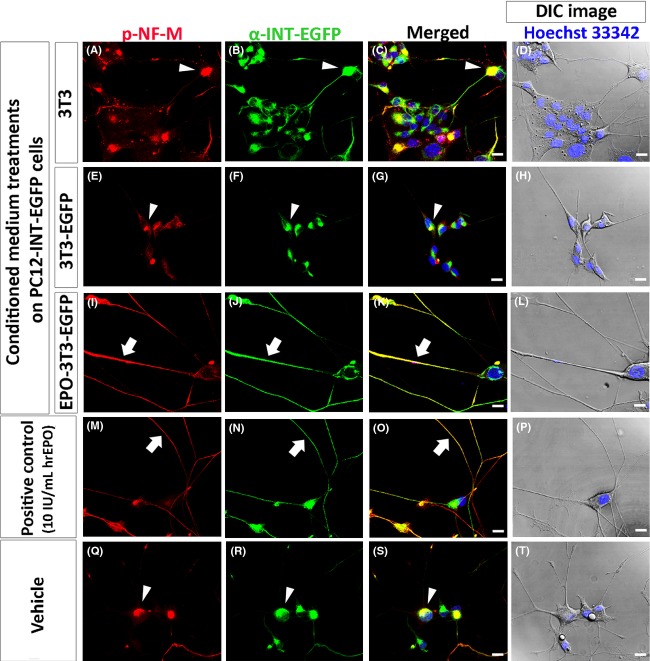
Distribution patterns of p-NF-M and *α*-INT-EGFP in PC12-INT-EGFP cells after 3T3, 3T3-EGFP, and EPO-3T3-EGFP conditioned media treatments. PC12-INT-EGFP cells were fixed at 24 h after supplementation with 3T3 (A–D), 3T3-EGFP (E–H), and EPO-3T3-EGFP (I–L) conditioned media, or with 10 IU/mL of hrEPO (M–P) and the vehicle (Q–T). p-NF-M and *α*-INT-EGFP appeared to be aggregated and mainly located in the perikaryal region of PC12-INT-EGFP cells after supplementation with 3T3 (A–C) and 3T3-EGFP (E–G) conditioned media, or the vehicle(Q–S). The merged images show that a large proportion of aggregated p-NF-M and *α*-INT-EGFP are colocalized (C, G, S, arrowheads; yellow). Surprisingly, p-NF-M (I, arrow) and *α*-INT-EGFP (J, arrow) in PC12-INT-EGFP cells supplemented with EPO-3T3-EGFP conditioned medium were mainly located in the neurite, and the merged image shows almost 100% colocalization (K, arrow; yellow) of p-NF-M and *α*-INT-EGFP. Similar patterns of distribution were also found in PC12-INT-EGFP cells supplemented with 10 IU/mL hrEPO (M–O, arrow), which was used as the positive control. DIC images were taken for each group (D, H, L, P, T), to depict cellular morphology. Scale bars = 20 *μ*m.

PC12-INT-EGFP cells were fixed and double immunostained with primary antibodies against NF-M or p-NF-M, together with *α*-INT-EGFP, respectively, at 24 h after supplementation with 3T3 (Figs.3A–C, 4A–C), 3T3-EGFP (Figs.3E–G, 4E–G), and EPO-3T3-EGFP (Figs.3I–K, 4I–K)-conditioned media, or with 10 IU/mL of hrEPO (Figs.3M–O, 4M–O) or the vehicle (Figs.3Q–S, 4Q–S). DIC images merged with Hoechst 33342 were also taken in each subgroup (Figs.3D, H, L, P and T, 4D, H, L, P and T), to depict cellular morphology. We observed that, in the groups supplemented with 3T3 (Figs.3A and B, 4A and B) and 3T3-EGFP (Figs.3E and F, 4E and F) conditioned media, or with the vehicle (Figs.3Q and R, 4Q and R), NF-M, p-NF-M, and *α*-INT-EGFP appeared to be aggregated and mainly located in the perikaryal region. The merged images indicated that a large proportion of aggregated NF-M, p-NF-M, and *α*-INT-EGFP were colocalized (Figs.3C, G and S, 4C, G and S; arrowhead; yellow).

Surprisingly, we observed that NF-M (Fig.3I; arrow), p-NF-M (Fig.4I; arrow), and *α*-INT-EGFP (Figs.3J, 4J; arrow) were transported into neurites and appeared to be less aggregated in the perikaryal region of PC12-INT-EGFP cells supplemented with EPO-3T3-EGFP conditioned medium and that the merged images showed high colocalization of either NF-M and *α*-INT-EGFP or p-NF-M and *α*-INT-EGFP (Figs.3K, 4K; arrow; yellow). Similar distribution patterns were also found in PC12-INT-EGFP cells supplemented with 10 IU/mL of hrEPO (Figs.3M–O, 4M–O; arrow), which were used as the positive control.

Conclusively, rearranged and disaggregated patterns of NF-M, p-NF-M, and *α*-INT-EGFP, as assessed by immunocytochemical analysis in PC12-INT-EGFP cells, could be found in the groups supplemented with EPO-3T3-EGFP-conditioned medium and 10 IU/mL of hrEPO.

## Discussion

Neurotrophins, such as NGF, brain-derived growth factor (BDNF), neurotrophin-3 (NT-3), NT-4, and EPO, are key regulators of the proliferation, maturation, and axonal regeneration of neurons (Tabira et al. 1995; Bibel and Barde 2000; Chao et al. 2006; Logan et al. 2006). Studies have shown that 3T3 fibroblasts secrete NGF (Young et al. 1975) and BDNF (Chen et al. 2012). However, the production of EPO in 3T3 fibroblasts has not been demonstrated in previous studies. In our study, the characteristic examinations of NIH/3T3 fibroblast showed little expression level of EPO; nevertheless, EPO-3T3-EGFP cells exhibited a significantly high expression and secretion levels of EPO as demonstrated in Fig.1 and Table 2010. Therefore, we concluded that the expression of endogenous EPO in NIH/3T3 cells was below the threshold of detection. After the overexpression of EPO in NIH/3T3 cells, large amount of cytosolic EPO was secreted into the extracellular milieu.

hrEPO at 10 IU/mL has been proven to be sufficient for an optimal neuroprotective effect regarding neuronal survival in vitro (Sirén et al. 2001; Wu et al. 2007; Cho et al. 2011). Hence, to examine and compare the bioactivity of the EPO secreted from EPO-3T3-EGFP cells, which was quantitated as 2.7 ng/mL in conditioned medium (50% v/v), 10 IU/mL of hrEPO (equal to 86.7 ng/mL of hrEPO) was also applied as a positive control in functional assays. Interestingly, although a 40-fold lower amount of secreted EPO from EPO-3T3-EGFP cells was present in cells treated with the conditioned medium compared with hrEPO, our results demonstrated the existence of similar neuroprotective effects on degenerating PC12-INT-EGFP cells after supplementation with either EPO-3T3-EGFP conditioned medium or 10 IU/mL of hrEPO. To further clarify the relationship of dosage and neuroprotective effectiveness of the EPO-3T3-EGFP conditioned medium compared with hrEPO, we applied 10 IU/mL of hrEPO as well as 0.25 IU/mL of hrEPO, which had the same amount of EPO as quantified in the EPO-3T3-EGFP conditioned medium, to the 3T3 conditioned medium. Our results showed that the significant difference could be observed between the groups supplemented with 0.25 IU/mL of hrEPO plus 3T3 conditioned medium and 10 IU/mL of hrEPO plus 3T3 conditioned medium; whereas no significant difference was found between the groups supplemented with 3T3 conditioned medium and 0.25 IU/mL of hrEPO plus 3T3 conditioned medium (Fig. S2). Therefore, we could further confirm that the EPO-3T3-EGFP conditioned medium had the equivalent neuroprotective effectiveness as 10 IU/mL of hrEPO. Therefore, the cell dosage will be carefully tested before conducting the in vivo studies.

In our study, the immunocytochemical patterns of NF-M showed that this protein was mainly located in the perikaryal region and appeared to be aggregated in the vehicle group or the groups supplemented with conditioned media from 3T3 and 3T3-EGFP cells. (Fig.3A, E and Q). Conversely, after supplementation with EPO, we found that NF-M was less aggregated in the perikaryal region and was transported into neurites (Fig.3I and M). As the role of nonphosphorylated NFs in axonal transportation has been well studied (Hoffman 1995; Chevalier-Larsen and Holzbaur 2006), we expected that the aberrant axonal transportation observed in PC12-INT-EGFP cells might be recovered after supplementation with EPO. Recently, it was reported that the motile phase of phosphorylated NFs not only processes the turnover of NFs but also mediates self-association to promote axonal outgrowth by cross-linked phosphorylated NFs (Barry et al. 2007; Shea and Lee 2011). Therefore, phosphorylated NFs promote axonal outgrowth, whereas nonphosphorylated NFs mediate axonal transportation (Hoffman 1995; Barry et al. 2007; Shea and Lee 2011). According to our immunostaining results, the distribution patterns of p-NF-M in PC12-INT-EGFP cells supplemented with EPO showed that the protein was mainly located in the processes (Fig.4I and M). In addition, previous studies have shown that neurotrophins are internalized from distal axons and transported retrogradely to the cell body, which is thought to be critical for cell survival (Ye et al. 2003; Wagner et al. 2004; Chevalier-Larsen and Holzbaur 2006). Retrograde transport is mediated by dynein, which binds directly to NF-M (Ye et al. 2003; Chevalier-Larsen and Holzbaur 2006). Our immunocytochemistry results showed that NF-M and p-NF-M were transported into neurites and became less aggregated in the perikaryal region and indicated that the neuroprotective ability of EPO might overcome perikaryal aggregation, restore axonal transportation, reduce cellular toxicity, and, consequently, enhance neuronal survival (Fig.2).

Our real-time cell imaging provided reliable and powerful information for tracing the distribution of fluorescent *α*-INT-EGFP in PC12-INT-EGFP cells after conditioned media treatments. We found progressive rearrangement and disaggregation of some *α*-INT-EGFP in the groups supplemented with the conditioned medium from EPO-3T3-EGFP cells or with 10 IU/mL of hrEPO (Fig. S3 and Supplementary Videos). The disaggregation ability of EPO has been investigated in ALS cell models overexpressing mutant SOD1 (Cu/Zn-binding superoxide dismutase) in NSC-34 cells (Cho et al. 2011). However, the mechanism underlying the disaggregation effect of EPO on cytoskeletons remains unclear and should be investigated further in the future.

More importantly, we established an EPO-overexpressing NIH/3T3 fibroblast cell line, EPO-3T3-EGFP, that produced an abundant amount of EPO and showed an effective neuroprotective effect of enhancing neuronal survival. Therefore, this EPO-overexpressing NIH/3T3 fibroblast cell line may represent a potential material for future studies of cell-based therapies for neurological diseases via the secretion of EPO on a short-term, high-dose, and regional basis.

## Supporting Information

**Video S1.** Time-lapse_3T3 conditioned medium treatment.wmv.

**Video S2.** Time-lapse_3T3-EGFP conditioned medium treatment.wmv.

**Video S3.** Time-lapse_ EPO-3T3-EGFP conditioned medium treatment.wmv.

**Video S4.** Time-lapse_ positive control_hrEPO.wmv.

**Video S5.** Time-lapse_ vehicle.wmv.

**Figure S1.** Live-cell imaging and immunocytochemical staining of 3T3, 3T3-EGFP, and EPO-3T3-EGFP stable cell clones. **Figure S2.** Cell-viability assay for cultured PC12-INT-EGFP cells after 3T3, 0.25 IU hrEPO + 3T3, 10 IU hrEPO + 3T3, and 3T3-EGFP conditioned media treatments for 48 h. **Figure S3.** 48 h live-cell imaging of PC12-INT-EGFP cells after 3T3, 3T3-EGFP, and EPO-3T3-EGFP conditioned media treatments.
