# Supplementary material for: Erythropoietin produced by genetic-modified NIH/3T3 fibroblasts enhances the survival of degenerating neurons
Source: Brain Behav. 2015 Jun 3;5(8):e00356. doi: 10.1002/brb3.356 (PMC4559019; doi:10.1002/brb3.356)
Supplement: Supplementary file 6 [file brb30005-e00356-sd6.docx]

## Supplementary Videos. 48h live-cell imaging of PC12-INT-EGFP cells after conditioned media treatments

Time-lapse microscopy images were acquired every 20 min, and transformed into videos for 10 images/s for 48 h to examine morphological changes and the dynamic distribution of the overexpressed green fluorescent α-internexin-EGFP fusion protein (α-INT-EGFP) in PC12-INT-EGFP cells after supplementation with different conditioned media. We observe that aggregated green fluorescent α-INT-EGFP proteins become much larger, and more dying PC12-INT-EGFP cells appear in the groups supplied with 3T3 (**Video 1**), 3T3-EGFP (**Video 2**) conditioned media, and the vehicle group (**Video 5**). Surprisingly, it can be found that a fraction of aggregated α-INT-EGFP dynamically rearranged, disaggregated and transported into the neurites of PC12-INT-EGFP cells after supplied with EPO-3T3-EGFP conditioned medium (**Video 3**) and hrEPO (**Video 4**). Cells also look healthier in the groups supplemented with EPO-3T3-EGFP conditioned medium and hrEPO. hrEPO (10 IU/mL) was applied as the positive control.

**Video 1: Time-lapse_ 3T3 conditioned medium treatment.wmv**

**Video 2: Time-lapse_ 3T3-EGFP conditioned medium treatment.wmv**

**Video 3: Time-lapse_ EPO-3T3-EGFP conditioned medium treatment.wmv**

**Video 4: Time-lapse_ positive control_hrEPO.wmv**

**Video 5: Time-lapse_ vehicle.wmv**


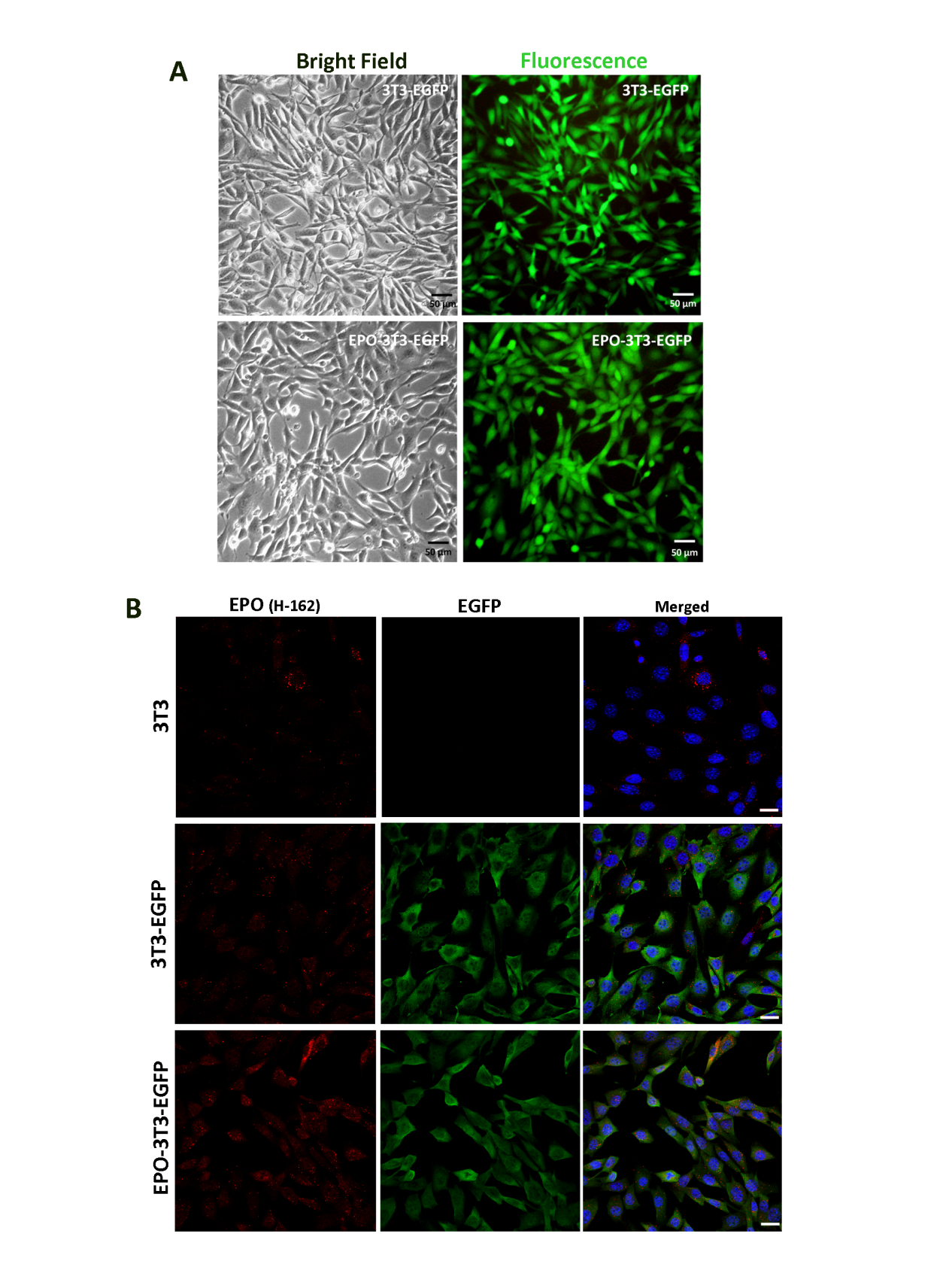


## Figure S1. **Live-cell imaging and immunocytochemical staining of 3T3, 3T3-EGFP, and EPO-3T3-EGFP stable cell clones** (A) Live-cell images of established 3T3-EGFP and EPO-3T3-EGFP stable cell clones are examined by phase-contrast and fluorescence microscopy using an inverted fluorescence microscope. Constitutively expressed EGFP was observed in both stable cell clones after G418 stable clone selection. Scale bars = 50 μm. (B) The 3T3, 3T3-EGFP, and EPO-3T3-EGFP cell lines were also double stained with anti-EPO and anti-EGFP antibodies. Although a few cells with immunopositive staining for endogenous EPO were found in the 3T3 cell group, rather stronger immunopositive staining of EPO was observed in EPO-3T3-EGFP cells compared with 3T3 and 3T3-EGFP cells. Immunopositive staining for EGFP was detected in both 3T3-EGFP and EPO-3T3-EGFP cells. Scale bars = 20 μm.

**A**

**
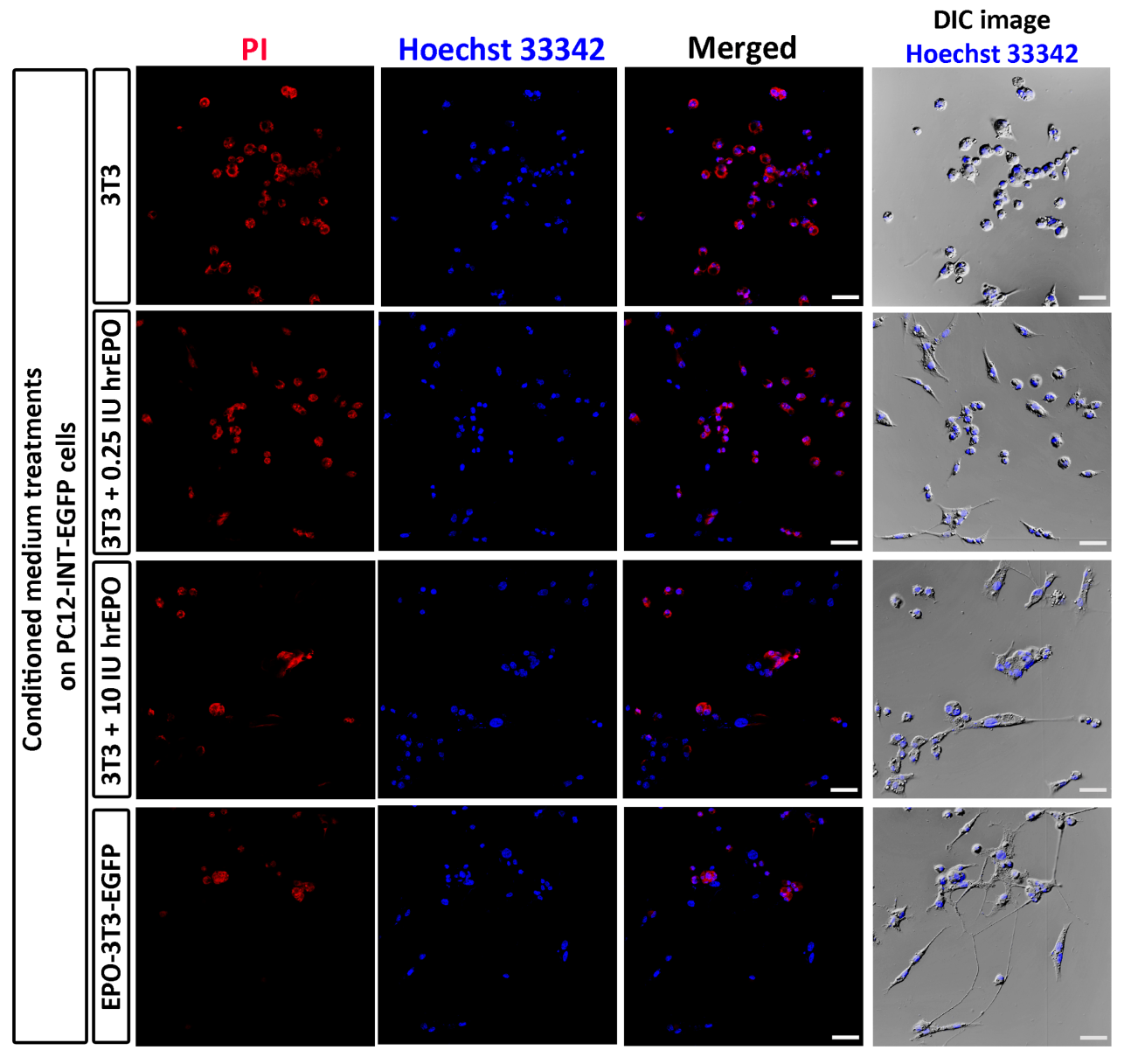
**

**B**

**
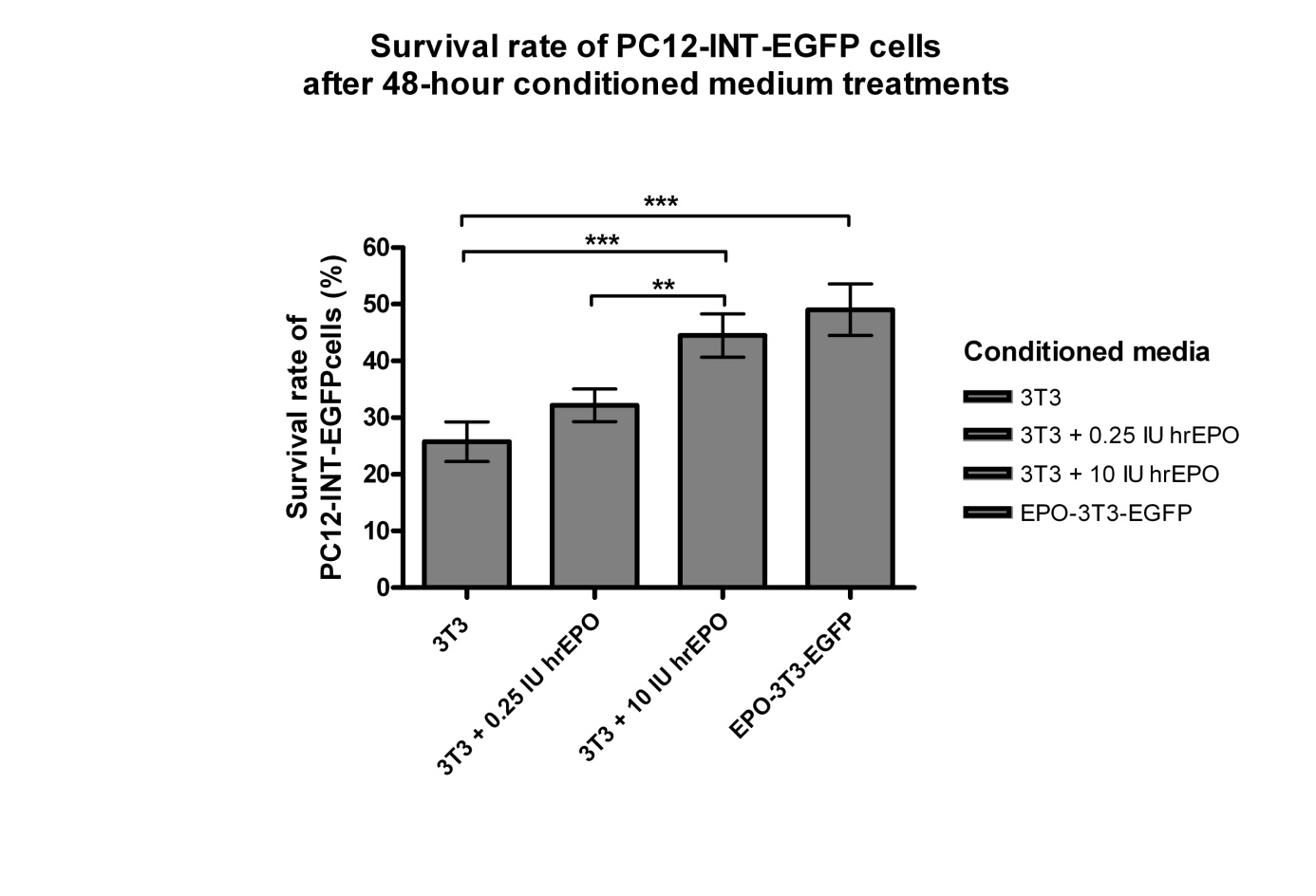
**

**(Conditioned media)**

## Figure S2. Cell-viability assay for cultured PC12-INT-EGFP cells after 3T3, 0.25 IU hrEPO + 3T3, 10 IU hrEPO + 3T3, and 3T3-EGFP conditioned media treatments for 48 h

To further clarify the relationship of dosage and neuroprotective effectiveness of the EPO-3T3-EGFP conditioned medium compared with hrEPO, we applied 10 IU/mL of hrEPO as well as 0.25 IU/mL of hrEPO, which had the same amount of EPO as quantified in the EPO-3T3-EGFP conditioned medium, to the 3T3 conditioned medium. We conducted the cell viability assay for the survival of PC12-INT-EGFP cells was assessed using (A) PI/Hoechst 33342 costaining of cellular nuclei after supplementation with 3T3, 0.25 IU hrEPO + 3T3, 10 IU hrEPO + 3T3, and 3T3-EGFP conditioned media for 48 h after 6 days of NGF induction. Scale bars = 40 μm.

(B) The survival rate of PC12-INT-EGFP cells supplemented with 10 IU hrEPO + 3T3 (44.49% ± 3.80%, mean ± SD, n = 4) and EPO-3T3-EGFP conditioned medium (49.04% ± 4.55%, mean ± SD, n = 4) was significantly enhanced compared with that observed in the groups supplemented with 0.25 IU hrEPO + 3T3 (32.19% ± 2.88%) and 3T3 (25.78% ± 3.52%) conditioned media. No significant difference was found between the groups supplemented with 3T3 and 0.25 IU hrEPO + 3T3 conditioned medium. Interestingly, the significant difference could be observed between the groups supplemented with 0.25 IU hrEPO + 3T3 and 10 IU hrEPO + 3T3. Therefore, we could confirm that the EPO-3T3-EGFP conditioned medium had the equivalent neuroprotective effectiveness as 10 IU/mL of hrEPO. (one-way ANOVA, ***P* < 0.01, ****P* < 0.001).

**
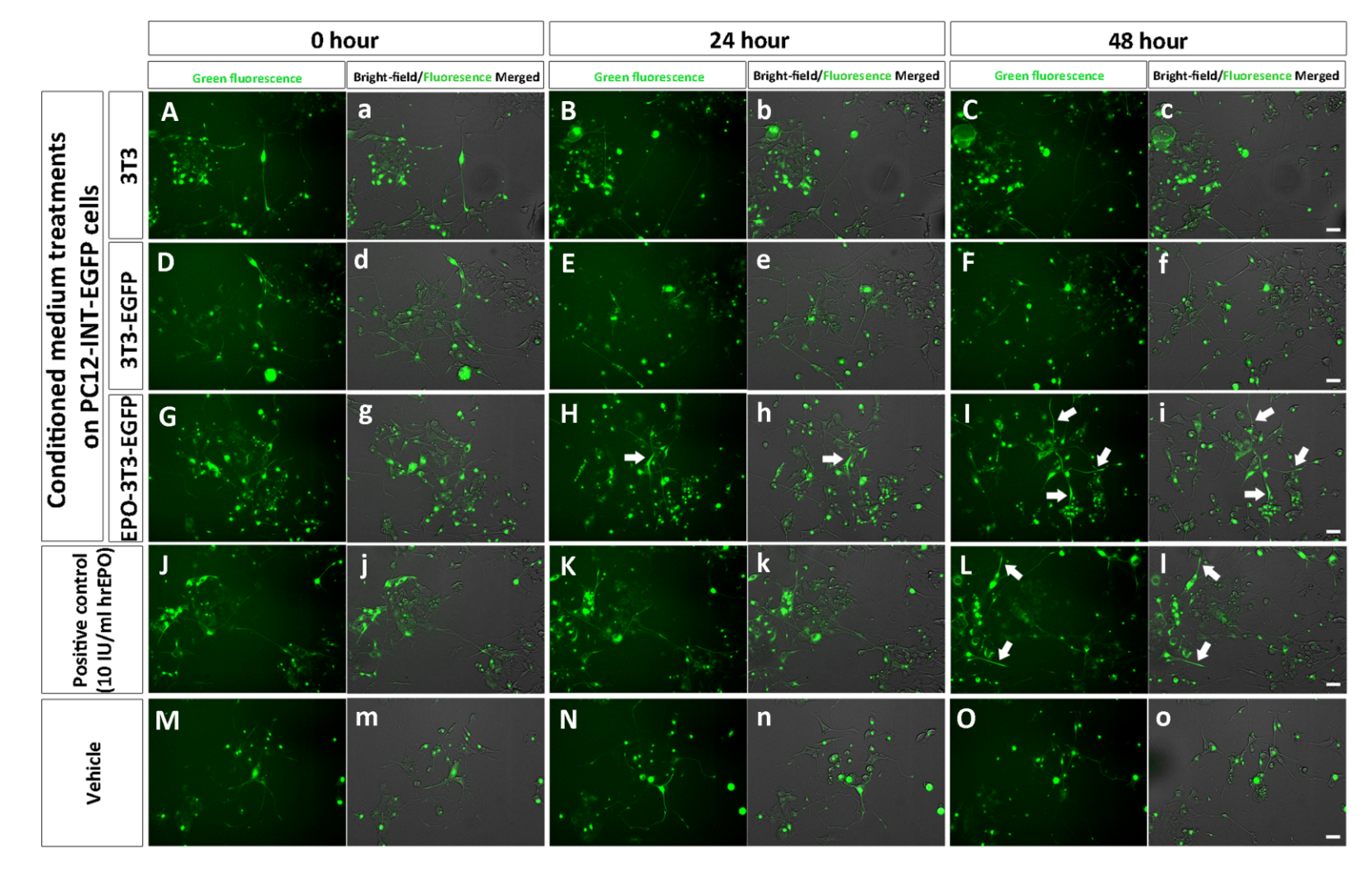
**

## Figure S3. 48h live-cell imaging of PC12-INT-EGFP cells after 3T3, 3T3-EGFP, and EPO-3T3-EGFP conditioned media treatments Morphological changes and the distribution of the overexpressed α-internexin-EGFP fusion protein (α-INT-EGFP) in PC12-INT-EGFP cells were recorded by time-lapse microscopy for 48 h after supplementation with conditioned media (50% v/v) collected from the 3T3 (A–C and a–c), 3T3-EGFP (D–F and d–f), and EPO-3T3-EGFP (G–I and g–i) cell groups on day 6 after NGF induction. hrEPO (10 IU/mL) (J–L and j–l) was used as the positive control. The comparison of green fluorescence images as well as bright fields merged with green fluorescence images at 0, 24, and 48 h after conditioned media treatments revealed that some aggregated green fluorescent α-INT-EGFP proteins in PC12-INT-EGFP cells exhibited dynamic patterns of disaggregation and were transported into neurites after supplementation with conditioned medium from EPO-3T3-EGFP cells (H, h, I, i, arrows) but not after supplementation with the conditioned media from 3T3 and 3T3-EGFP cells or from the vehicle group (M–O and m–o). A few PC12-INT-EGFP cells supplemented with 10 IU/mL of hrEPO also showed similar patterns of disaggregation (L and l, arrows). Scale bars = 50 μm.
